# Antimicrobial mechanisms of nanopatterned surfaces—a developing story

**DOI:** 10.3389/fchem.2024.1354755

**Published:** 2024-01-29

**Authors:** Arash Pirouz, Ioannis Papakonstantinou, Martyna Michalska

**Affiliations:** ^1^ Manufacturing Futures Lab, Department of Mechanical Engineering, University College London, London, United Kingdom; ^2^ Photonic Innovations Lab, Department of Electronic and Electrical Engineering, University College London, London, United Kingdom

**Keywords:** bioinspired, biomimetic, biofouling, antimicrobial mechanisms, mechanobactericidal surfaces, nanopatterns, driving force

## Abstract

Whilst it is now well recognized that some natural surfaces such as seemingly fragile insect wings possess extraordinary antimicrobial properties, a quest to engineer similar nanopatterned surfaces (NPSs) is ongoing. The stake is high as biofouling impacts critical infrastructure leading to massive social and economic burden with an antimicrobial resistance (AMR) issue at the forefront. AMR is one of the most imminent health challenges the world is facing today. Here, in the effort to find more sustainable solutions, the NPSs are proposed as highly promising technology as their antimicrobial activity arises from the topographical features, which could be realized on multiple material surfaces. To fully exploit these potentials however, it is crucial to mechanistically understand the underlying killing pathways. Thus far, several mechanisms have been proposed, yet they all have one thing in common. The antimicrobial process is initiated with bacteria contacting nanopatterns, which then imposes mechanical stress onto bacterial cell wall. Hence, the activity is called “mechano-bactericidal”. From this point on, however, the suggested mechanisms start to diverge partly due to our limited understanding of force interactions at the interface. The aim of this mini review is to analyze the state-of-the-art in proposed killing mechanisms by categorizing them based on the characteristics of their driving force. We also highlight the current gaps and possible future directions in investigating the mechanisms, particularly by shifting towards quantification of forces at play and more elaborated biochemical assays, which can aid validating the current hypotheses.

## Introduction

Antimicrobial resistant microorganisms are now one of the top 10 global public health threats according to the World Health Organization. To tackle the AMR challenges, several domestic and global action plans have been developed, largely focused on identifying effective and feasible infection prevention and control measures. Here, bioinspired nanopatterns with antimicrobial properties are considered particularly promising as they can kill bacteria (and other microorganisms) or inhibit their proliferation by virtue of their topography (Linklater et al., 2021; Hawi et al., 2022). The fact that antimicrobial activity stems from the topography arguably enables them to be applied on any surface where biofouling is a problem (by direct surface structuring or application of nanostructured film). Thus, the potential applications of antimicrobial NPSs are widespread spanning from public transportation, mobile displays, water purification systems, packaging, to personal protecting equipment and medical implants (Ganjian et al., 2019; Valiei et al., 2020; Michalska et al., 2021b).

Conventional antimicrobial surfaces rely on diffusion of pre-immobilized biocides, which has certain drawbacks. These include compound degradation, reduced potency over time, uncontrolled release, and, importantly, inefficiency against ever-growing AMR bacteria (Andersson and Hughes, 2014; Cloutier et al., 2015). Though it is noteworthy that antimicrobial NPSs do not comprehensively resolve all these challenges; for example, the reduced efficiency over time and pattern durability remained the major bottlenecks awaiting to be resolved (Wang et al., 2020; Hoque et al., 2023). Nevertheless, the unique topographically triggered antimicrobial activity of these surfaces has drawn significant research interest, and understanding how these surfaces operate is the first research question yet to be fully answered (Lin et al., 2018).

Since the first report on antibacterial properties of natural (Ivanova et al., 2012) and synthetic substrates (Ivanova et al., 2013), researchers have tried to elucidate the underlying killing mechanisms. The prevalent opinion has been that the mechanical interactions and force exchanges between the NPSs and bacteria at the bio-interface compromises the cell wall integrity, which in turn leads to the loss of internal turgor pressure and eventual death. However, the origin of forces acting on the bacterial cell wall and, consequently, its failure mode are still unsettled. For instance, the physical damage was firstly attributed to cell wall uniaxial strain beyond its elastic limit over the suspended region (Pogodin et al., 2013). Alternatively, over the following years, various and often contradictory failure causes have been proposed, whose driving force arises from different sources such as adhesion (Michalska et al., 2018), stored elastic energy (Ivanova et al., 2020), cell motility (Bandara et al., 2017), and capillary forces (Valiei et al., 2020).

Of note, whether rupturing the cell wall is the primary cause of cell death is being additionally questioned. In fact, a recent study on stochastic TiO_2_ nanopillars (pillars are one example of nanoprotrusions; the notation is used throughout the text for simplicity) reports that bacterial cell wall undergoes localized deformation, which leads to only occasional penetration. Hence, it cannot account for all the observed reduction in viability (Jenkins et al., 2020). This observation alongside complementary biological assays—a recent trend in the field, have prompted a new take on the killing mechanisms. Namely, it postulates that the mechanical stimuli imparted on bacterial envelop could also be transduced to a chemical signal, leading to oxidative stress inside the cells, which eventually can activate apoptosis-like pathways (Zhao et al., 2022; Le et al., 2023). In other words, antimicrobial NPSs may have the capacity to induce gene and proteomic alteration inside the cells. Thus, the antimicrobial properties could also be attributed to a biochemical response to the cell wall perturbances. However, the exact pathways by which the mechanical signal is transduced into a chemical signal that creates redox imbalance inside the cells remains unclear.

The difficulty in reaching a consensus over the killing mechanisms stems from multiple challenges pertinent to small scales of both topography and bacteria, which makes real-time direct observations or force measurements difficult or impossible (Lin et al., 2018; Bandara et al., 2020; Valiei et al., 2020). Lack of universal protocols to evaluate the performance and attention to small but important details like, e.g., presence of air bubbles during testing, further complicates the issue and comparison of the designs (Valiei et al., 2020; Michalska et al., 2021a). Additionally, many highly efficient patterns are random or heterogenous, which makes it harder to delineate the performance boundaries. Overall, it is likely that a combination of mechanisms accounts for the cell death rather than just one. Nevertheless, investigating the mechanisms one at a time, if possible at all, is insightful in terms of determining their contribution in the final bacterial death. Also, establishing the killing mechanism(s) paves the way for further improvements such as increasing the killing efficiency and pattern durability. The latter is equally important as the properties remain as long as the pattern is present, which is, however, beyond the scope of this review. The readers may refer to some recent advances in this regard reported by Yu et al. (2021), Zhang et al. (2021). Here, we firstly review the purely mechanical mechanisms classified by the proposed driving forces, followed by examination of the mechanically triggered mechanisms.

## Mechanical mechanisms

To establish that NPSs are “mechano-bactericidal,” it must be proven there are active force exchanges at the bacteria-substrate interface, sufficient to damage the cell wall leading to death either directly or indirectly (mechanical or mechanically triggered mechanisms, respectively). The damage of the cell walls occurs when sufficient mechanical stress, whose minimum magnitude depends on the cell wall mechanical properties, is imparted on the cell wall. To describe mechano-bactericidal mechanisms, the first step is identifying the driving forces that could govern these interactions. Several forces of different origins have been proposed and investigated via simulations and/or experimentally where possible, which can be grouped as follows: 1) non-specific interactions, 2) stored-elastic energy release, and 3) capillary forces.

## Non-specific interactions

**FIGURE 1:**
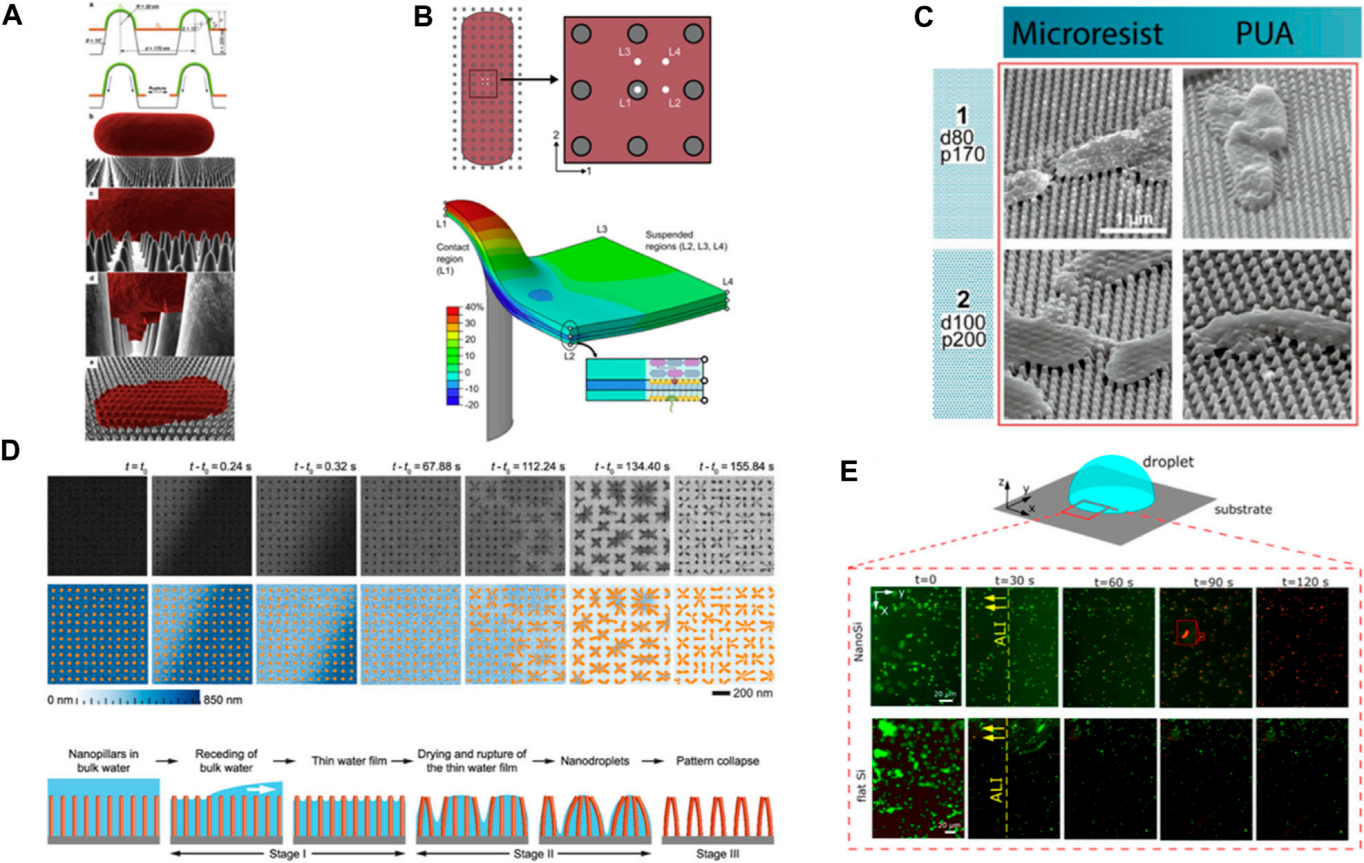
**(A)** Schematic of different stages of bacterial interaction with nanopillars based on biophysical model. The adsorbed layer increases its surface area which leads to the cell wall rupture over suspended region between nanopillars. Reprinted with permission from Pogodin et al. (2013). Copyright 2023 Elsevier. **(B)** Contour plot illustrates the longitudinal uniaxial strain of a multi-layer bacterial envelop adhered on a nanopillar. The plot reveals a nonuniform strain distribution in different layers and locations, with a critical site of action identified at the apex of the nanopillar. Reprinted with permission from Velic et al. (2021). Copyright 2023 Elsevier. **(C)** SEM images of *E. coli* cells interaction with deflected nanopillars. Reprinted with permission from Lohmann et al. (2022). Copyright 2023 American Chemical Society. **(D)** (Top) Time-resolved *in situ* TEM image series showing elastocapillary action during water evaporation from high AR silicon nanopillars surfaces. (Bottom) Schematic illustration of different stages of the dewetting process. Reprinted with permission from Vrancken et al. (2020). Copyright 2023 American Chemical Society. **(E)** *P. aeruginosa* viability on nanopillars (NanoSi) and control (flat) Si surfaces upon evaporation. Viability of bacteria as a function of time on NanoSi and flat Si that is subject to evaporation (live cells are green and dead cells are red). Reprinted with permission from Valiei et al. (2020). Copyright 2023 American Chemical Society.

The “biophysical model” by Pogodin et al. (2013) was the first proposed mechanism to explain the bactericidal action of cicada wings. According to this model, deformational stress distributes non-uniformly across the cell wall at the adsorption sites and the suspended region. It assumes that stress is developed by bacterial cells’ tendency to maximize their adsorption area and minimize the gained free energy through cell wall deformation. Since the nanopillars are more accessible as the adsorption sites, it is more likely that the cell wall ruptures over the suspended region (Figure 1A). Conversely, a recent finite element analysis of a Gram-negative cell on cicada-like surfaces suggests that critical nondevelopable strain occurs at the pillar apex (Figure 1B) (Velic et al., 2021). Likewise, observations from various imaging techniques reveal the critical site of action is rather the protrusions’ top (Linklater et al., 2017; Jenkins et al., 2020).

The driving force in both models is thought to be nonspecific intermolecular adhesion forces (Van der Waals, hydrogen bonding, electrostatic) with the biophysical model not specifying their magnitude. In the computational model by Velic et al. (2021) on the other hand, the data used to compute adhesion energy lacks an appropriate experimental validation. Here, methods like Single Cell Force Spectroscopy (SCFS) could provide the relevant data. For example, various groups indicate the magnitude of nonspecific interaction forces between Gram-positive/negative bacteria and relatively smooth hydrophilic/hydrophobic silicon substrates to be of the order of a few nN (Potthoff et al., 2015; Beaussart et al., 2020). This value is expected to decrease upon nanostructuring due to the reduction in effective surface area, as reported for *Staphylococcus* (S.) aureus on nanopillars (Doll et al., 2022). The quantitative approaches in these studies are inspiring indeed for designing future experiments to determine the role of nonspecific forces at the interface.

## Stored-elastic energy release

Scanning electron microscopy (SEM) images of bacteria on high aspect ratio (AR) nanopillars from various studies (conducted for 15 min–18 h) revealed that peripheral nanostructures deflect towards the cells (Figure 1C) (Bandara et al., 2020; Ivanova et al., 2020). This observation has prompted researchers to investigate the impact of nanopillars flexural rigidity (i.e., a combination of materials elastic modulus and AR) on killing efficiency of NPSs. For certain patterns, an enhanced killing efficiency is reported and attributed to the release of stored elastic energy of pillars upon deflection that acts as an external force onto bacterial cell wall (Linklater et al., 2018). From these efficient patterns, we learn that depending on nanopillars’ elastic modulus, geometrical features and their deflection, the calculated force needed to deform each pillar varies from ∼10 nN (Ivanova et al., 2020) to 200–500 nN (Lohmann et al., 2022). Despite the thicker geometries in the latter case, the significantly lower elastic modulus of the studied materials when compared to silicon in the former case makes one wonder how realistic these values are. Nevertheless, we consider this range and postulate that is not entirely clear whether bacteria cells can generate such force, to deflect approximately 10 pillars around their cell body. Bearing in mind the challenges to compute these values however, like ambiguity whether elastic modulus changes at nanoscale at all or how nanoscale defects affect the results, we attempt to discuss it in the context of available forces at play. The already described intermolecular adhesion forces act instantaneously and as such, we deem them irrelevant here. However, we consider three probable scenarios including forces generated by 1) migrating bacteria, 2) extracellular polymeric substances (EPS) (Bandara et al., 2017), and 3) capillary action.

We first look at the magnitudes of generated forces during bacteria migrations. To migrate, cells must exert forces, and this is dependent at minimum on the motility type and relevant appendages or their lack. Although little is known about the possibility of bacteria migration on NPSs, the studies in this field yield information about the level of “strong” forces possible to be exerted. For example, *Neisseria* gonorrhea’s individual type IV pili generate 100 pN, while the pili bundles’ force does not exceed a few nN (Marathe et al., 2014). Single Molecular Force Spectroscopy data suggests similar results for *Pseudomonas* (P.) aeruginosa’s type IV pili (Dufrêne et al., 2021). In another study, a single cell and collective migration of Myxococcus xanthus were analyzed by Traction Force Microscopy, and revealed for the latter case that the exerted force is below 300 pN (Sabass et al., 2017). Hence, it appears that bacterial force generation apparatus alone may not exert sufficient force to be directly responsible for nanopillars’ deflection.

For the bacterial cells interacting at longer times, the role of secreted extracellular polymeric substance (EPS) cannot be excluded. Here, forces up to 30 nN have been recorded during live imaging of Xylella fastidiosa adhered on protein coated-InP nanowires for 24 h (Sahoo et al., 2016). Considering the surface coating and bacteria dwelling time, it can be argued that the interactions are indeed EPS-mediated. If this is the case however, one may think of EPS-mediated force transfer because the energy stored in pillars may not be in direct contact with the bacterial cells anymore. Of note, the bacteria models above mostly vary from the ones typically used in mechano-bactericidal studies. One of the potential future directions may include closing the gap of force generation capabilities by different strains.

Although trivial at large scales, capillary forces might become dominant at sub-micrometer scale. In fact, capillary forces may deform solid bodies, a phenomenon called elastocapillary. In the field of nanomanufacturing, collapse of high AR nanostructures has been extensively studied and reported for solution-based processing (Chandra and Yang, 2009; Mallavarapu et al., 2020; Michalska et al., 2023). Real-time monitoring of water evaporation from an array of Si nanopillars by using *in situ* liquid phase Transmission Electron Microscopy (TEM) revealed that as the water evaporates, discrete nanodroplets form, ultimately leading to nanopillars’ collapse (Figure 1D). Depending on whether the interpillar attraction forces are greater than the elastic-stored energy or not, the pillars remain clustered or revert to their initial position (Vrancken et al., 2020). Therefore, capillary forces could be potentially responsible for the deflections when surface tension is high and there is a case for bacteria-NPSs-liquid-air interface. Although we do not know how the presence of adhered bacteria on nanopillars changes the dynamics of capillary forces, SEM images suggest that bacteria may amplify the clustering (Figure 1C). While some of the discussed studies consider the issue and use, e.g., critical point drying during sample preparation for microscopy to exclude it, others do not, which might contribute to an effect. More about the impact of capillary action is described below.

## Capillary forces

Capillary forces are present at bacteria cell-substrate interface when the level of liquid falls below the bacterial cell height. Since capillary forces are usually dominant over other surface forces, they are a sensible candidate to suspect as driving force for pulling bacteria towards the nanoprotrusions and damaging the cell wall. In fact, Valiei et al. (2020) reported lack of killing on hydrophilic silicon nanopillars even after 24 h of incubation while cells were still fully immersed in the medium, i.e., no capillary force was present at the interface. However, when the liquid droplet starts to evaporate, a live/dead staining of the cells at the periphery of the moving air-liquid interface revealed remarkable >90% of killing only after 2 min (Figure 1E). The findings in this study indicate that capillary forces are large enough to create downward force and kill bacteria (Valiei et al., 2020). Most likely, harnessing this effect could dramatically increase the antimicrobial property of NPSs, albeit it depends on surface energy and topography. Moreover, at any stage of exposing bacteria to the NPSs, the development of capillary forces may act as an external and dominant force to kill bacteria. Thus, careful experimental operations are suggested if one desires to distinguish between capillary action- or other force-induced killings.

## Mechanically triggered mechanisms

**FIGURE 2:**
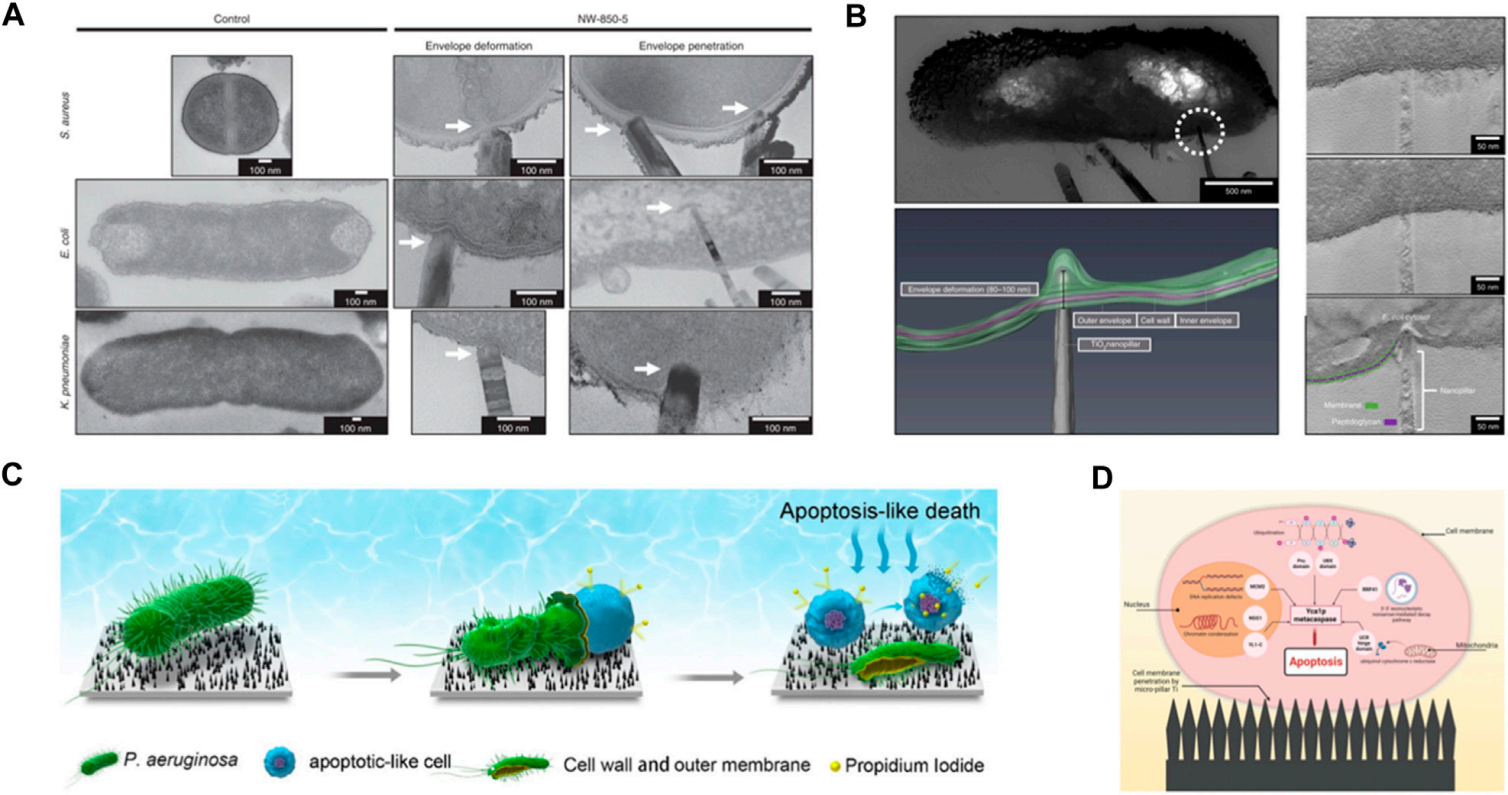
**(A)** TEM micrographs of TiO_2_ nanopillars-mediated bacterial envelope deformation and penetration in Gram-positive and Gram-negative bacteria. Adapted from Jenkins et al. (2020). **(B)** Tomographic reconstruction of nanopillar-induced envelop deformation in *E. coli*. Adapted from Jenkins et al. (2020). **(C)** Schematic illustration of death process of *P. aeruginosa* cell in contact with bSi nanospikes. Adapted with permission from Zhao et al. (2022). Copyright 2023 American Chemical Society. **(D)** Schematic depiction of how mechanically triggered mechanism induces microorganism apoptosis. Mechanical injuries as the result of interactions with nanopillars activate pathways that lead to programmed death. Reprinted from Le et al. (2023).

The interactions at bacteria-NPSs interface captured by imaging techniques inspired the field to propose new mechanisms or challenge the long-lasting beliefs (Figure 2A). For example, 3D visualization of bacteria-TiO2 nanopillar interactions, reconstructed from electron tomography, suggests that *Escherichia* (E.) coli’s cell wall can be locally deformed around the pillars as much as 190 nm without being ruptured (Figure 2B). The study indicates that penetration events are infrequent, and they cannot explain the reported reduction in viability assays (Jenkins et al., 2020). These observations, alongside complementary biological assays, have prompted a new take on how NPSs operate—antimicrobial properties of NPSs may not have a purely physical nature. Instead, it has been suggested that the physical signals (mechanical stress) received by bacterial cell wall can be transduced to bio/chemical signals that could impair internal processes such as bacterial growth (Jenkins et al., 2020) or activate apoptosis-like death pathways (Zhao et al., 2022).

Analyses of proteomic profiles of *E. coli* and *S. aureus* after 24 h incubation on nanopillars show that differentially expressed proteins in both strains are biologically connected, implying a generic response across bacterial strains. Furthermore, the data indicate that some of these proteins are associated with cell response to elevated reactive oxygen species (ROS) production, further confirmed by measuring H_2_O_2_ levels—an oxidative stress marker. Therefore, the reduction in bacteria viability here has been attributed to the activation of ROS-dependent lethal pathways (Jenkins et al., 2020).

A recent investigation of mechano-bactericidal property of black silicon (bSi) nanopillars suggests that a combination of mechanically injured cell wall, internal turgor pressure, and a weak coupling between the cell wall and plasma membrane allows the cytoplasmic content of *P. aeruginosa* to leak out while cells are viable (Figure 2C). Subsequent exposure of the fluid membrane to nanopillars increases the intracellular ROS production, which activates apoptosis-like death (Figure 2D) (Zhao et al., 2022). Even though the mediating biochemical links between each of these stages are not apparent yet, the occurrence of these events is independently supported by electron microscopy images and biological assays.

## Discussion

Understanding the mechanism(s) behind the bactericidal action of NPSs underlies the basis for their application. The key to unraveling the role of each mechanism itself is understanding its driving force and, ideally, quantifying it. Simultaneous fluorescence imaging and nanoindentation of *E. coli* with atomic force microscopy showed that force of 25 nN can puncture the cell wall to kill almost 90% of a cell population (Del Valle et al., 2020). While such quantification is a good reference, one needs to consider the highly localized character of the measured force. Having this in mind, we now attempt to review the magnitude of the proposed driving forces as a quantitative approach in assessing the efficacy of the killing mechanisms. Mechano-microbiological studies indicate that the magnitude of forces generated by bacterial varies between several hundreds of pN (Sabass et al., 2017; Dufrêne et al., 2021) and tens of nN for longer dwelling times in a presence of EPS (Sahoo et al., 2016). On the other hand, studies that investigated the non-specific interactions reported the forces from 1 nN (Doll et al., 2022) to 40 nN (Maikranz et al., 2020) for nanopillars and relatively smooth Si surfaces, respectively. These results imply that the reduction of effective surface area on nanopillars would further undermine the nonspecific interaction role as dominant driving force for cell death.

Capillary action arising from surface tension at the physical boundary of different phases at the bacteria-NPSs-liquid-air interface is suggested to play a critical role in creating downward forces strong enough to damage bacteria cell wall (Valiei et al., 2020). A recent study further confirmed that the morphology of *P. aeruginosa* cells incubated on superhydrophilic Si nanopillars under continuous wet conditions showed no sign of damage to the cell wall as visualized by SEM, after specimen fixing and critical point drying. When the same samples were prepared for the SEM after water was evaporated, cell morphology was visibly destroyed (Valiei et al., 2022). Considering that capillary forces are only introduced when the liquid level recedes a certain threshold, it is not easy to measure the force experimentally. Nevertheless, there is enough evidence in literature to show that capillary action plays a dominant role at the air/liquid interface of nano/microstructures (Chandra and Yang, 2009; Mallavarapu et al., 2020).

The deflection of nanopillars around bacteria cell body has been widely reported and is believed to contribute to increase the mechano-bactericidal efficiency by exerting the stored elastic energy on the cell wall (Linklater et al., 2018; Ivanova et al., 2020; Lohmann et al., 2022). Here, we argue that forces generated by the cells may not be sufficient to deflect nanopillars within the reported range of 10 nN–500 nN (Lohmann et al., 2022). To the best of our knowledge, currently there is no compelling evidence to support that cells can generate this amount of force on such surfaces. To illuminate the observations, it seems more studies are needed to both quantify the forces exerted by bacteria and attempt to measure and/or simulate with more accuracy the force required to deflect nanopillars. It was also suggested that perhaps we should look at stress instead of absolute force (Lohmann et al., 2022). Perhaps, particularly for the higher reported forces, a careful verification of sample preparation for SEM imaging would be beneficial to exclude a contribution of capillary action. The more extensive discussion around this mechanism in this review stems from the appreciation of complexity to validate its assumptions. While we debate the force magnitude exerted by bacteria under “passive” adhesive conditions, the capillary action is one example of added external force to yield an enhanced killing. Others not discussed here and overall, less explored may include, e.g., shear, acoustic, or magnetic forces. Conversely, under those “passive” conditions, it is plausible that cells experience sublethal damage, which leads us to a discussion of mechanically triggered mechanisms.

Here, both cited groups reveal quite complex mechanisms, which cannot be captured by standard characterization techniques employed in the field, namely, colony counting and live/dead fluorescent staining. The latter technique seems to be the most frequently utilized in the field, often without the former method. These studies hence highlight that interpretation of such results can be grossly misleading, potentially underscoring a real activity/value of some already reported surface designs. In fact, this issue had been already pointed out by Michalska et al. (2021a), where the discrepancies in bactericidal performance were similarly noted when flow cytometry technique was employed, albeit on the loosely attached cells. Importantly for practical applications, while (Zhao et al., 2022), reveal that sublethal injured cells can undergo post-stress ALD when transferred to a non-stressed environment, in contrast, Michalska et al. (2021a) show that some injuries can be repaired. In this aspect, the major differences between the studies are interaction time and “the non-stressed environment,” where phosphate saline buffer and super optimal broth with catabolite repression medium were used, respectively.

Most certainly, post-stress research needs more focus in the future to deepen the mechanistic understanding but also to provide relevant information to practical applications. While quantitative studies on interacting forces are also needed as well as more biochemical investigations, another future challenge appears to be exploring the factors that make one mechanism dominate over the others. These surely include topography, the interfacing mode between bacteria and the surfaces ( ± external force, etc.), and the kind of bacteria species. This would enable true control over the interactions’ mechanics. This, in turn, could be then leveraged to design strategies for more efficient killing as well as harness other untapped opportunities in the field.
